# Immunomodulatory Effect of Marine Cembrane-Type Diterpenoids on Dendritic Cells

**DOI:** 10.3390/md11041336

**Published:** 2013-04-22

**Authors:** Ching-Yen Lin, Mei-Chin Lu, Jui-Hsin Su, Ching-Liang Chu, David Shiuan, Ching-Feng Weng, Ping-Jyun Sung, Kao-Jean Huang

**Affiliations:** 1 Department of Life Science and Institute of Biotechnology, National Dong Hwa University, Hualien 974, Taiwan; E-Mails: jouyuan22@gmail.com (C.-Y.L.); shiuan@mail.ndhu.edu.tw (D.S.); cfweng@mail.ndhu.edu.tw (C.-F.W.); 2 Graduate Institute of Marine Biotechnology, National Dong Hwa University, Pingtung 944, Taiwan; E-Mails: jinx6609@nmmba.gov.tw (M.-C.L.); x2219@nmmba.gov.tw (J.-H.S.); pjsung@nmmba.gov.tw (P.-J.S.); 3 Graduate Institute of Immunology, College of Medicine, National Taiwan University, Taipei 100, Taiwan; E-Mail: clchu01@ntu.edu.tw

**Keywords:** antigen presentation, bone-marrow derived dendritic cell (BMDC), marine natural compound, tumor necrosis factor-α

## Abstract

Dendritic cells (DCs) are antigen presenting cells, which can present antigens to T-cells and play an important role in linking innate and adaptive immunity. DC maturation can be induced by many stimuli, including pro-inflammatory cytokines and bacterial products, such as lipopolysaccharides (LPS). Here, we examined the immunomodulatory effects of marine cembrane compounds, (9*E*,13*E*)-5-acetoxy-6-hydroxy-9,13-dimethyl-3-methylene-3,3a,4,5,6,7,8,11,12,14a-decahydro-2*H*-cyclotrideca[*b*]furan-2-one (**1**), (9*E*,13*E*)-5-acetoxy-6-acetyl-9,13-dimethyl-3-methylene-3,3a,4,5,6,7,8,11,12,14a-decahydro-2*H*-cyclotrideca[*b*]furan-2-one (**2**), lobocrassin B (**3**), (−)14-deoxycrassin (**4**), cembranolide B (**5**) and 13-acetoxysarcocrassolide (**6**) isolated from a soft coral, *Lobophytum crassum*, on mouse bone marrow-derived dendritic cells (BMDCs). The results revealed that cembrane-type diterpenoids, especially lobocrassin B, effectively inhibited LPS-induced BMDC activation by inhibiting the production of TNF-α. Pre-treatment of BMDCs with Lobocrassin B for 1 h is essential to prohibit the following activation induced by various toll-like receptor (TLR) agonists, such as LPS, zymosan, lipoteichoic acid (LTA) and Pam2CSK4. Inhibition of NF-κB nuclear translocation by lobocrassin B, which is a key transcription factor for cytokine production in TLR signaling, was evident as assayed by high-content image analysis. Lobocrassin B attenuated DC maturation and endocytosis as the expression levels of MHC class II and the co-stimulatory molecules were downregulated, which may affect the function of DCs to initiate the T-cell responses. Thus, lobocrassin B may have the potential in treatment of immune dysregulated diseases in the future.

## 1. Introduction

Marine compounds have emerged as the new era in discovering new therapeutic drugs, including those can regulate the inflammatory responses [1,2,3]. Recently, chemical investigations on soft coral *Lobophytum crassum* have led to the isolation and identification of varieties of oxygenated cembrane-type diterpenoids [4,5,6,7,8]. The *in vitro* assay model for screening compounds with anti-inflammatory activity was conducted in lipopolysaccharide (LPS)-treated RAW 264.7 and assayed for the expression levels of pro-inflammatory proteins, particularly the inducible nitric oxide synthase (iNOS) and cyclooxygenase-2 (COX-2). Some cembranoids have been shown to have anti-inflammatory effects by downregulating the iNOS and COX-2 expression [9,10,11,12]. In addition, sinularin, a cembrane-type diterpenoid isolated from *Sinularia* sp., exerted its bioactivities on anti-inflammatory effect, as well as the analgesic property both *in vitro* and *in vivo*. In LPS-stimulated RAW cells, sinularin significantly inhibits iNOS and COX-2 expression and upregulates the production of transforming growth factor-β (TGF-β), which may contribute to the suppression of microglial and astrocyte activation in carrageenan-induced tissue inflammatory responses [13].

Dendritic cells (DCs) are the professional antigen-presenting cells, which are important in linking the innate and adaptive immunity [14]. DC maturation can be induced by many stimuli, including pro-inflammatory cytokines and bacterial products, such as LPS. Immature DCs bear the pattern recognition receptors (PRRs) for microbes, such as toll-like receptors (TLRs), possess endocytosis ability to load antigens and then migrate toward the draining lymph nodes, where they mature and present the proteasome-degraded antigens to naive T-cells [15]. Mature DCs stop antigen-loading, but enhance the expression of major histocompatibility complexes (MHC) and accessory molecules, such as CD86, CD80 and CD40, and then produce cytokines and chemokines [16]. The signaling cascade responsible for LPS-stimulated DCs is involved the TLR4 binding lipopolysaccharide (LPS), p38 mitogen-activated protein kinases (MAPKs) activation and Nuclear Factor-kappa B (NF-κB) activation [17]. In general, NF-κB is retained in cytoplasm and complexes with the inhibitor of κB (I-κB) repressor protein. Upon activation by potent stimuli, *i.e.*, LPS, I-κB is phosphorylated by I-κB kinase (IKK) and subsequently degraded by ubiquitin-mediated proteasomal degradation and NF-κB dissociated from I-κB in cytoplasm and then translocated into nucleus to activate the inflammatory cytokine and chemokine genes [18]. Thus, DCs play the role in controlling infectious diseases, as well as cancers [19]. However, DCs also participated in the pathogenesis of several immune dysregulated diseases, including chronic inflammation and autoimmunity [20,21]. Intervention of such disease progression may be achieved by suppression or attenuation of DC activation.

In this study, we examined the immuno-regulatory effect of cembrane-type diterpenoids on bone marrow-derived dendritic cells (BMDCs). Our results showed that lobocrassin B may be a potent immunosuppressant to inhibit DC maturation and activation, suggesting that lobocrassin B may have therapeutic applications in certain immune dysfunctions.

## 2. Results and Discussion

### 2.1. Suppression of TNF-α Expression in BMDCs by Cembrane-Type Diterpenoids

**Figure 1:**
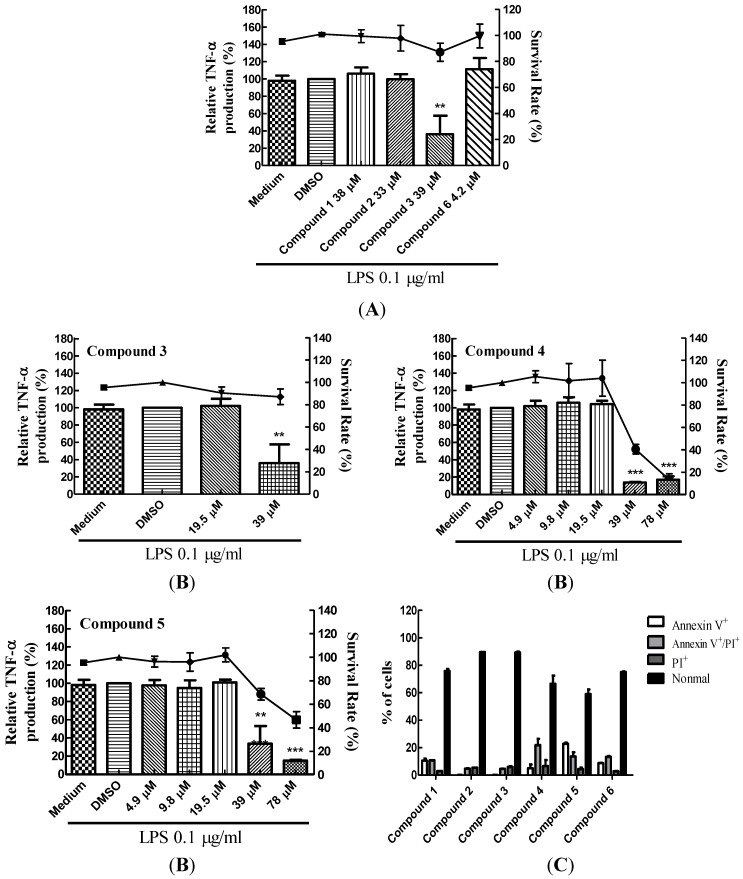
Suppression of TNF-α production in lipopolysaccharides (LPS)-stimulated BMDCs by cembrane-type diterpenoids. (**A**) Inhibition of TNF-α production in BMDCs by coral cembranolides. BMDCs (2 × 10^5^/well) in 96-well plates were untreated or treated with 0.1 μg/mL of LPS with individual cembranolides (**1**, **2**, **3** (lobocrassin B) or **6**) at a concentration of CC_20_ for 6 h, and mTNF-α levels in the culture supernatant were measured by ELISA. Cell toxicity evaluated by the MTT method was also included. (**B**) Dose-effects of lobocrassin B (**3**) and lobocrassin B-related compounds (**4** and **5**) on inhibition of TNF-α production in LPS-stimulated BMDCs. BMDCs (2 × 10^5^/well) were untreated or treated with 0.1 μg/mL of LPS plus indicated concentrations of lobocrassin B (**3**), **4** and **5** for 6 h, and mTNF-α levels in the culture supernatant were measured by ELISA. The relative mTNF-α production (%) in each group was normalized to the LPS-stimulated control (LPS plus DMOS). Bars represent the relative mTNF-α production, and lines represent the survival rate. The data present as the mean ± SD. ** *p* < 0.01 *vs.* LPS-stimulated control; *** *p* < 0.001 *vs.* LPS-stimulated control. (**C**) Direct cytotoxicity of compound **1**–**6** on LPS-stimulated BMDCs. BMDCs incubated with compound **1**–**6** at the concentration used in (**A**) for 6 h were harvested and stained with PI and Annexin V-fluorescein isothiocyanate (FITC) for flow cytometry. The percentage of apoptotic cells (Annexin V^+^ and Annexin V^+^ PI^+^), dead cells (PI^+^) and survival cells (Annexin V^−^ PI^−^) were calculated and plotted.

The cembrane-type metabolites were isolated from soft octocoral, *Lobophytum crassum*, and the cytotoxic effects of these compounds on BMDCs were assayed by the MTT method. Among them, Compound **6** was more toxic to BMDCs, with the cytotoxicity (CC_20_) about 4.2 μM. The others were less toxic, as their CC_20_ were 6–9-fold higher than Compound **6** (Table 1). These compounds were tested for their potency to affect the function of BMDCs by measuring TNF-α production. Compounds **1**–**6** did not induce TNF-α production in immature BMDCs (data not shown), but lobocrassin B (**3**) at CC_20_ concentration had the potency to suppress the TNF-α production from LPS-stimulated BMDCs (Figure 1A). We further tested compounds **4** and **5**, which are structurally similar to lobocrassin B for their activities to suppress the TNF-α production. Compounds **4** and **5** could suppress TNF-α production, but they caused more cell growth inhibition (60 and 30%, respectively) at the same concentration (39 μM) as used for lobocrassin B (Figure 1B). The direct cell cytotoxicity caused by compound **1**–**6** was further confirmed by Annexin V-propidium iodide (PI) staining after treatment with individual compounds for 6 h. The direct cytotoxicity mediated by lobocrassin B was 10.7%, and this was less toxic than 33.5, 41 and 25% for Compound **4**, **5** and **6**, respectively (Figure 1C). Hence, lobocrassin B could be a potent suppressor that can inhibit TNF-α production from LPS-activated BMDCs, and compounds structurally similar to lobocrassin B may also exert the same effect, but to a different extent.

### 2.2. Blocking of Various TLR Agonists-Mediated TNF-α Production in BMDCs by Lobocrassin B

**Figure 2:**
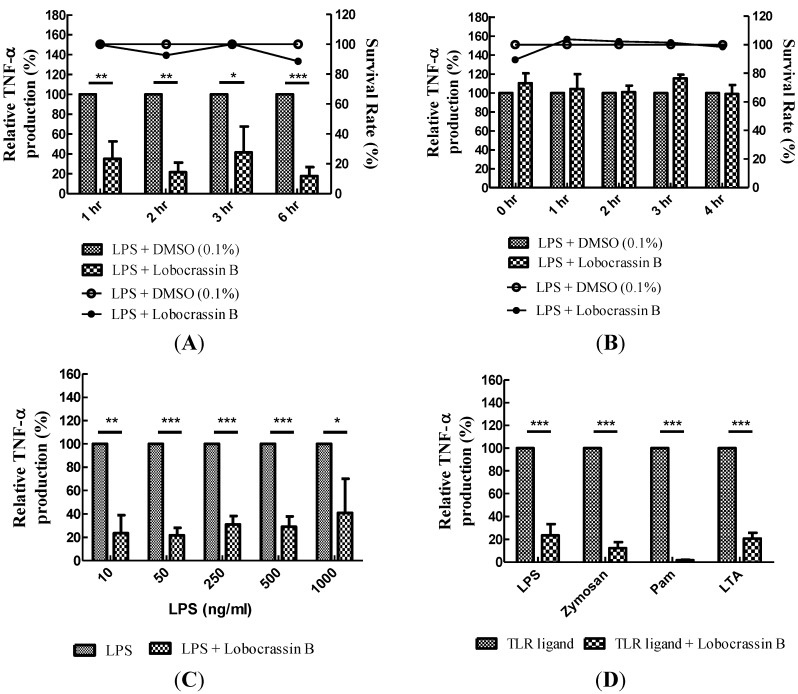
Inhibitory effect of lobocrassin B on various TLR agonists-stimulating TNF-α production. (**A**) Pre-treatment of BMDCs with lobocrassin B (39 μM) before LPS stimulation. (**B**) Post-treatment of LPS-stimulated BMDCs with lobocrassin B. (**C**) Inhibitory capacity of lobocrassin B on LPS-stimulated BMDCs. BMDCs were pre-treated with lobocrassin B (39 μM) for 1 h and followed by incubating with increasing dosages of LPS (10, 50, 250, 500 and 1000 ng/mL) for a total 6 h. Cell culture supernatants were collected at 6 h after LPS stimulation, and TNF-α levels were measured by ELISA. The data present as the mean ± SD. * *p* < 0.05 *vs.* LPS-stimulated control; ** *p* < 0.01 *vs.* LPS-stimulated control; *** *p* < 0.001 *vs.* LPS-stimulated control. (**D**) Inhibitory effect of lobocrassin B on various TLR ligands-stimulating BMDCs. BMDCs were pre-treated with lobocrassin B (39 μM) for 1 h and then stimulated with LPS (100 ng/mL), Zymosan (20 μg/mL), Pam (20 ng/mL) and LTA (20 μg/mL). Cell culture supernatants were harvested at 6 h, and TNF-α levels were measured by ELISA. The data present as the mean ± SD. *** *p* < 0.001 *vs.* individual TLR ligand-stimulated control.

As lobocrassin B is effective in blocking TNF-α production in LPS-stimulated BMDCs, we further confirmed the duration for lobocrassin B’s effect. Pre-treatment of lobocrassin B on BMDCs as short as 1 h was enough to inhibit TNF-α production from LPS-stimulated BMDCs (Figure 2A), while post-treatment had no effect on the inhibition of TNF-α production (Figure 2B), indicating that lobocrassin B may act on the upstream of LPS-stimulating signaling. As LPS is a potent activator for BMDCs, once its signaling was initiated, lobocrassin B may not be able to counteract this vast impact.

The capacity of lobocrassin B for inhibiting the LPS-stimulated TNF-α production was evaluated. Lobocrassin B could suppress the TNF-α production of BMDCs when they were stimulated by LPS at as high concentration as 1000 ng/mL (Figure 2C). In addition, we tested whether lobocrassin B could exert the same inhibitory effect when BMDCs were stimulated by various TLR agonists other than LPS. BMDCs were stimulated by Zymosan (for TLR2), Pam (for TLR2) or LTA (for TLR2), and the suppression of TNF-α production for each was evident, where the inhibitory effects by lobocrassin B for each agonists were comparable to that of LPS, *i.e.*, equal or less than 20% in TNF-α production. Thus, lobocrassin B could be used as a blocker acting on the early events in TLR-related signaling (Figure 2D).

**Table 1:** The cytotoxic effect of marine cembrane-type diterpenoids on murine bone marrow-derived dendritic cells (BMDCs).

Compound	Structure	Molecular Weight	CC_20_ (μM) *
(9*E*,13*E*)-5-Acetoxy-6-hydroxy-9,13-dimethyl-3-methylene-3,3a,4,5,6,7,8,11,12,14a-decahydro-2*H*-cyclotrideca[*b*]furan-2-one (**1**)	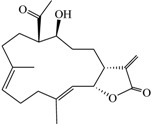	332.43	38
(9*E*,13*E*)-5-Acetoxy-6-acetyl-9,13-dimethyl-3-methylene-3,3a,4,5,6,7,8,11,12,14a-decahydro-2*H*-cyclotrideca[*b*]furan-2-one (**2**)	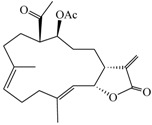	374.47	33
Lobocrassin B (**3**)	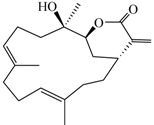	318.45	39
(−)14-Deoxycrassin (**4**)	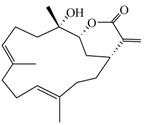	318.45	26.9
Cembranolide B (**5**)	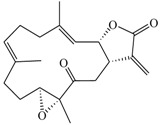	330.42	36
13-Acetoxysarcocrassolide (**6**)	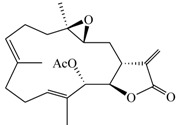	374.47	4.2

* Cell cytotoxicity test in murine BMDCs was determined by MTT assay.

### 2.3. Dose-Dependent Inhibition of TNF-α Production and NF-κB (p65) Nuclear Translocation by Lobocrassin B

**Figure 3:**
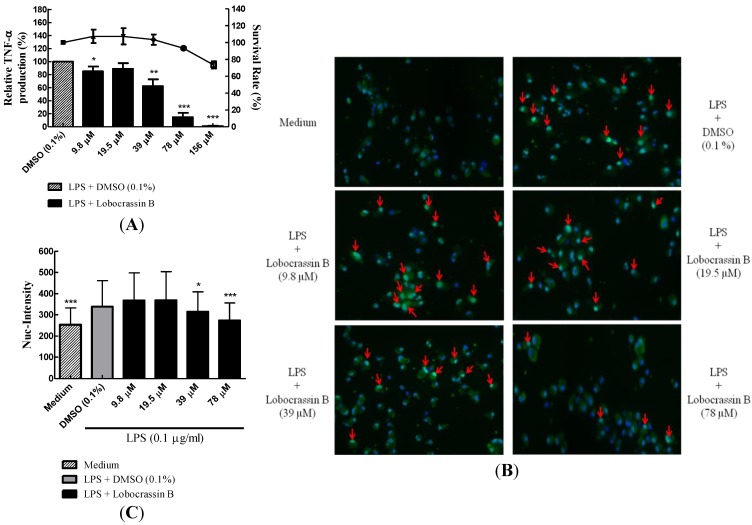
Dose-dependent inhibition of TNF-α production and NF-κB (p65) nuclear translocation in LPS-stimulating BMDCs by lobocrassin B. (**A**) Dose-dependent inhibition of TNF-α production by lobocrassin B. BMDCs were pre-treated with indicated concentrations of lobocrassin B (9.8, 19.5, 39, 78 and 156 μM) for 1 h and then followed by stimulation with 0.1 μg/mL of LPS for 5 h. Culture supernatants were collected, and TNF-α production was assayed by ELISA. (**B**) Dose-dependent inhibition of NF-κB (p65) nuclear translocation by lobocrassin B. BMDCs were pre-treated with various doses of lobocrassin B as in (**A**) for 1 h and then stimulated with 0.1 μg/mL of LPS for 5 h. Cells were fixed with 3.7% formaldehyde and stained with anti-NF-κB (p65) RabMAb followed by the Alexa Fluor 488-conjugated anti-rabbit IgG (H+L) antibody. Cells were further counterstained with Hochest 33258, and images were captured and analyzed. The arrow indicates the nuclear localization of NF-κB. (**C**) High content image analysis of NF-κB nuclear translocation in (**B**). The fluorescence intensity of NF-κB within nucleus was analyzed by BD Attovision software. The data present as the mean ± SD. * *p* < 0.05 *vs.* LPS-stimulated control; ** *p* < 0.01 *vs.* LPS-stimulated control; *** *p* < 0.001 *vs.* LPS-stimulated control.

Activation of BMDCs by TLR agonists (e.g., LPS) can transduce signaling cascade involving the downstream NF-κB transcription factor that may dissociate from I-κB in cytoplasm and then translocate into the nucleus to activate cytokine and chemokine genes. The production of TNF-α was inhibited by lobocrassin B in a dose-dependent manner (Figure 3A), and the corresponding LPS-mediated NF-κB nuclear translocation was also attenuated as assayed by high content image analysis (Figure 3B,C). These results revealed that lobocrassin B indeed affects the signaling transduction mediated by TLRs.

### 2.4. Attenuation of LPS-Induced DC Maturation and Endocytosis by Lobocrassin B

**Figure 4:**
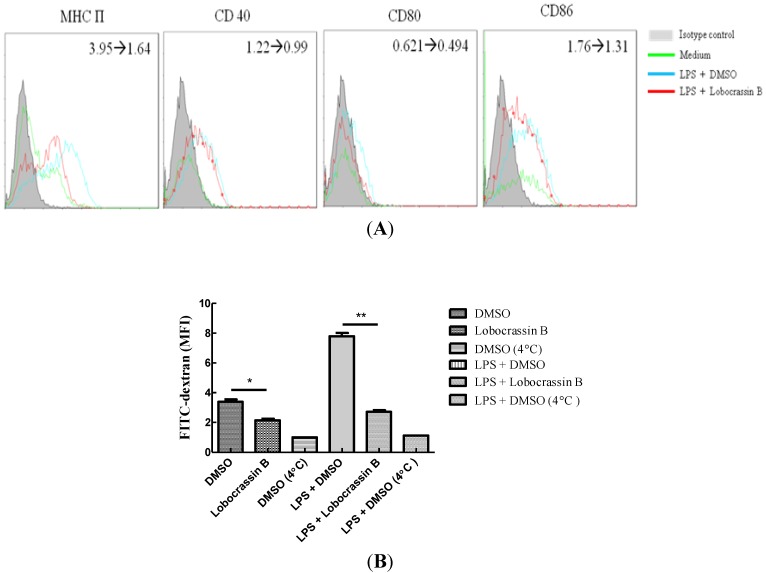
Lobocrassin B attenuated LPS-induced dendritic cell (DC) maturation and endocytosis. (**A**) Inhibition of BMDC maturation by lobocrassin B. BMDCs were treated with LPS (0.1 μg/mL) in 0.1% DMSO or LPS plus lobocrassin B (39 μM) for 24 h, and the expression levels of MHC II, CD40, CD80 and CD86 in each treatment were determined by flow cytometry. All data were gated on CD11c^+^ cells. (**B**) Blocking of BMDC endocytosis by lobocrassin B. BMDCs were pretreated with 0.1% DMSO or lobocrassin B (39 μM) for 1 h and then incubated with LPS (0.1 μg/mL) plus dextran-FITC for an additional 1 h. The uptake of dextran-FITC (MFI) by BMDCs was measured by flow cytometry. Negative control was conducted with BMDCs incubated with dextran-FITC at 4 °C. The data present as the mean ± SD. * *p* < 0.05 *vs.* DMSO control; ** *p* < 0.01 *vs.* LPS-stimulated control.

DCs stimulated by invading microbes may undergo a maturation process, which in turn are capable of initiating the adequate adaptive immune responses. To investigate the effect of lobocrassin B on DC maturation, we examined the expression of MHC II and costimulatory molecules, such as CD86, CD80 and CD40 in BMDCs, which represent the key phenotypes of DC maturation. The expression levels of MHC class II, CD86, CD80 and CD40 on BMDCs were increased (mean fluorescence intensity (MFI) = 3.95, 1.22, 0.62 and 1.76, respectively) when stimulated with LPS alone, but decreased (MFI = 1.64, 0.99, 0.49 and 1.31, respectively) as the treatment combined with LPS and lobocrassin B (Figure 4A). Additionally, we also examined if the antigen-loading ability of DCs was affected by lobocrassin B. Fluorescein isothiocyanate (FITC)-labeled dextran was incubated with BMDCs in the treatment of DMSO, lobocrassin B, LPS plus DMSO, LPS plus lobocrassin B and LPS at 4 °C. Levels of endocytosis were determined by flow cytometry. LPS could enhance the endocytosis of FITC-dextran by BMDCs, but lobocrassin B alone or combined with LPS substantially reduced the uptake of FITC-dextran. The inhibitory level of endocytosis exerted by lobocrassin B was comparable to those treated at 4 °C (Figure 4B). However, the level of reactive oxygen species (ROS) within BMDCs induced by LPS were not affected by lobocrassin B, and lobocrassin B alone failed to induce ROS production (data not shown).

### 2.5. Discussion

Soft corals (Coelenterata, Octocorallia and Alcyonaceae) are rich in steroids and terpenoids, and mostly, the isolated diterpenes are cembrane-type compounds [22]. In previous studies, many cembranes have been found to exhibit various biological activities, such as antitumor [9,23,24,25,26], anti-microbial [27,28,29] and anti-inﬂammatory activities [10,24,25,30,31]. Within those, the cembrane-type diterpenoids with anti-inflammatory activities may decrease the expression levels of iNOS or COX-2, and somewhat upregulate TGF-β in macrophages [13].

Here, we set up the experiments according to a previous report with minor modification, where quercetin, one of the most common flavonoids in the diet, exerted the immunosuppressive effect on dendritic cell activation and function [32]. We demonstrated that cembranolides isolated from soft coral can also suppress the activation and maturation of murine DCs. The expression levels of MHC class II, co-stimulatory molecules and adhesion molecules are lower in immature DCs, while they become high in mature DCs when inflammatory stimuli are engaged [33]. TLR receptors are engaged with the inflammatory stimuli on immature DCs and then transduce signaling to activate certain transcriptional factors (NF-κB and AP-1), which initiate cytokine production and DC maturation [34,35]. Our results show that pre-treatment of immature DCs with cembranolides (e.g., lobocrassin B) effectively inhibits TNF-α production and attenuates DC maturation after LPS stimulation. Although the suppressive mechanisms mediated by lobocrassin B may not be identical to quercetin, the effective dosage of lobocrassin B used in this study is 39 μM, which is comparable to that (50 μM) of quercetin reported previously [32].

Additionally, NF-κB nuclear translocation was also inhibited dose-dependently by lobocrassin B treatment. In particular, marine natural products have recently been recognized as a promising source of NF-κB inhibitors [36,37,38]. The cembrane-type diterpenoids isolated from soft corals (*Sarcophyton* sp. and *Sinularia* sp.) have been shown to inhibit both TNF-α-induced NF-κB-DNA binding, as well as TNF-α-induced I-κB degradation and nuclear translocation of p50/p65 [39]. As NF-κB is the downstream molecule along with various TLRs-mediated signaling pathways, targeting of this molecule has a promising effect when various TLR agonists are engaged. Our results showed that lobocrassin B can antagonize the actions of various TLR ligands, such as LPS, Zymosan, Pam or LTA, by inhibiting the TNF-α production and the NF-κB activation in activated DCs. Thus, lobocrassin B may act as a NF-κB inhibitor in accordance with the finding that cembrane-type diterpenoids may interfere in the action of NF-κB [39] and could reduce the expression levels of downstream iNOS and COX-2 in anti-inflammatory responses. Suppression of the ROS production induced by LPS may be one reason to decrease NF-κB activity [40]; however, it seems not to be the action by lobocrassin B, as the induced intracellular ROS was not affected after lobocrassin B treatment (data not shown). In this study, our results suggest that cembrane-type diterpenoids isolated from the soft coral, *Lobophytum crassum*, may have an immunomodulatory effect on DCs and needs to be further studied in the treatment of those immune dysregulated diseases in the future.

## 3. Experimental Section

### 3.1. Mice and Generation of DCs

Male mice aged at 6–8 weeks were purchased from the National Laboratory’s Animal Center (Taipei, Taiwan) and were kept in a temperature-controlled environment (22 °C) with 70% relative humidity under a 12 h light/dark cycle. The animal experiments were performed according to the “Guide for the Care and Use of Laboratory Animals” of the National Dong-Hwa University. Bone marrow-derived dendritic cells (BMDCs) were generated from C57BL/6 mice bone marrow, as described previously [41,42]. Briefly, bone marrow cells were cultured in RPMI 1640 medium containing 10% fetal bovine serum, 2 mM of l-glutamine (Gibco BRL, Grand Island, NY, USA), streptomycin-penicillin (Biowest, Nuaillé, France), 50 ng/mL of GM-CSF and IL-4 (PeproTech, Rocky Hill, NJ, USA) for seven days. Half of the culture medium was replaced by fresh complete medium every 2–3 days. On day 7, cells were harvested and assayed for CD11c expression (a DC specific marker) by staining with PE-conjugated anti-CD11c antibody (AbD serotec, Raleigh, NC, USA). The percentage of immature DCs (CD11c^+^) was determined by FC500 flow cytometer (Beckmen Coulter, Taipei, Taiwan), and immature DCs on average accounted for 70% of total bone marrow cells in each preparation.

### 3.2. Preparation of Cembrane-Type Diterpenoids

Cembrane-type diterpenoids were isolated from a soft coral, *Lobophytum crassum*, and the extraction procedure was described previously [5]. Their chemical structures were previously identified as (9*E*,13*E*)-5-Acetoxy-6-hydroxy-9,13-dimethyl-3-methylene-3,3a,4,5,6,7,8,11,12,14a-decahydro-2*H*-cyclotrideca[*b*]furan-2-one (**1**) [43], (9*E*,13*E*)-5-Acetoxy-6-acetyl-9,13-dimethyl-3-methylene-3,3a,4,5,6,7,8,11,12,14a-decahydro-2*H*-cyclotrideca[*b*]furan-2-one (**2**) [43], lobocrassin B (**3**) [5], (−)14-deoxycrassin (**4**) [44], cembranolide B (**5**) [45] and 13-acetoxysarcocrassolide (**6**) [46]. Pure cembrane-type diterpenoids (**1**–**6**) were dissolved in dimethyl sulfoxide (DMSO) (Sigma-Aldrich, St. Louis, MO, USA) as stock solutions and further diluted in serum-free RPMI 1640 medium. The concentration of DMSO used in all experiments was less than 0.1%.

### 3.3. Cytotoxicity Assay of Cembrane-Type Diterpenoids

Immature DCs were treated with various marine cembranolides in the absence or presence of 0.1 μg/mL LPS (Sigma-Aldrich) for 6 or 24 h and then incubated with 3-[4,5-dimethylthiazol-2-yl]-2,5-diphenyltetrazolium bromide (MTT, concentration 2.5 mg/mL) (Sigma-Aldrich, St. Louis, MO, USA) for 4 h. The formazan crystal in purple color was developed from tetrazolium (MTT) within cells by the action of mitochondrial succinate dehydrogenase and was extracted into DMSO. The optical density (OD) of absorbance at 570 nm was measured by EnSpire^®^ Multimode Plate Reader (PerkinElmer, Santa Clara, CA, USA). The survival percentage of each group was calculated by (OD_570_ of treatment/OD_570_ of controls) × 100%. A direct cytotoxicity assay was conducted using an Annexin V kit (Santa Cruz Biotechnology, Dallas, TX, USA), according to the manufacturer’s instructions. Apoptosis was determined by flow cytometry.

### 3.4. Measurement of TNF-α Production

BMDCs (2 × 10^5^/well in 96-well plate) were incubated with indicated marine cembranolides, as listed in Table 1, or TLR ligands, including LPS (100 ng/mL), zymosan (20 μg/mL), lipoteichoic acid (LTA) (20 μg/mL) (Sigma-Aldrich, St. Louis, MO, USA) and synthetic diacylated lipoprotein Pam2Cys-Ser-Lys4 (20 ng/mL) (InvivoGen, San Diego, CA, USA), and after 6 h, cell culture supernatants were collected and assayed for TNF-α levels by mTNF-α kit (eBioscience), according to the manufacturer’s instructions. TLR ligands were used to stimulate DCs [32]. The detection limit of mTNF-α is 10 pg/mL.

### 3.5. High Content Image Analysis of NF-κB Nuclear Translocation

BMDCs (5 × 10^4^/well in BD 96-well imaging plate) were pre-treated with lobocrassin B at the indicated concentration of 9.8, 19.5, 39 and 78 μM for 1 h and then stimulated with 0.1 μg/mL of LPS for 5 h. The cells were fixed by cold methanol (Macron Chemicals) for 15 min and then washed trice using phosphate buffered saline (PBS). Cells were then stained with anti-NF-κB (p65) RabMAb (1:400 diluted in PBS) (Epitomics) at 4 °C overnight and washed 3 times with PBS followed by staining with 4 μg/mL of secondary Alexa Fluor 488 goat anti-rabbit IgG (H + L) (Invitrogen, Carlsbad, CA, USA) for 30 min. Cells were counterstained with Hochest 33258 (2 μg/mL) (Invitrogen) and subjected to image analysis using BD pathway 435 image analyzer (Becton, Dickinson and Company, BD, Franklin, NJ, USA). The fluorescence intensity of NF-κB (p65) within cells was analyzed by BD software, Attovision, according to the software’s user manual. More than 200 cells were analyzed in each group.

### 3.6. Assay of DC Maturation and Endocytosis Activity

DC maturation was evaluated by the upregulation of MHC class II and the costimulatory molecule expression, as described previously [32]. BMDCs were treated with LPS (0.1 μg/mL) plus DMSO (0.1%) or LPS plus lobocrassin B (39 μM) for 24 h. Cells were harvested and stained with mAbs specific for mouse CD11c, I-A^b^, CD40, CD80, CD86 (AbD serotec), MHC-II (eBioscience, San Diego, CA, USA) and negative isotype control (AbD serotec and eBioscience). The mean fluorescence intensity (MFI) for each molecule was analyzed by flow cytometry. The endocytosis by BMDCs was estimated by dextran-FITC uptake, as described. BMDCs were treated with 0.1% DMSO, lobocrassin B (39 μM), LPS (0.1 μg/mL) plus DMSO or LPS plus lobocrassin B for 1 h and then incubated with dextran-FITC (MW ~77 kD, Sigma-Aldrich, St. Louis, MO, USA) for another 1 h. Cells were collected and labeled with PE-conjugated anti-mouse CD11c before acquisition. The uptake of dextran-FITC by BMDCs (CD11c^+^) was analyzed by flow cytometry. A negative control was conducted with cells incubated with dextran-FITC at 4 °C.

## 4. Conclusions

Here, we examine the effects of marine cembrane-type diterpenoids, especially lobocrassin B, on mouse DCs. Lobocrassin B alone did not stimulate TNF-α production from BMDCs, but effectively inhibited LPS-induced DC activation by inhibiting the production of TNF-α. Pretreatment with lobocrassin B prohibited the activation of BMDCs induced by LPS, while post-treatment exerted no obvious effects on TNF-α production. In addition, lobocrassin B exhibited a broad spectrum of inhibiting DC activation mediated by various TLR agonists, such as LPS (for TLR4), zymosan (for TLR2), LTA (for TLR2) and Pam2CSK4 (for TLR2). NF-κB, an important transcription factor responsible for cytokine production in the downstream of TLR signaling, was also affected by lobocrassin B, as its nuclear translocation upon activation was inhibited. Lobocrassin B also attenuated DC maturation and endocytosis, as the expression levels of MHC class II and the costimulatory molecules, such as CD80, CD86 and CD40, were decreased in treatments, which may attenuate the function of DCs to activate naive T-cells during the initiation of adaptive immune responses. Thus, the application of lobocrassin B in the treatment of immune dysregulated diseases will be further studied in the future.
